# Soybean root transcriptome profiling reveals a nonhost resistant response during *Heterodera glycines* infection

**DOI:** 10.1371/journal.pone.0217130

**Published:** 2019-05-24

**Authors:** Wenwen Song, Nawei Qi, Chen Liang, Fangmeng Duan, Honghai Zhao

**Affiliations:** Key Lab of Integrated Crop Pest Management of Shandong Province, College of Plant Health and Medicine, Qingdao Agricultural University, Qingdao, China; Osmania University, INDIA

## Abstract

*Heterodera glycines* (soybean cyst nematode, SCN) is one of the most devastating pathogens of soybean worldwide. The compatible and in compatible interactions between soybean and SCN have well documented. Nevertheless, the molecular mechanism of a nonhost resistant response in soybean against SCN infection remains obscure. Toward this end, a global transcriptional comparison was conducted between susceptible and resistant reactions of soybean roots infected by taking advantage of finding a new pathotype of SCN (SCN_T_). The soybean cultivar Lee, which exhibits resistant to SCN_T_ and susceptible to HG 1.2.3.4.7 (SCN_s_) was utilized in the expriments. The results highlighted a nonhost resistant response of soybean. Transcriptome analysis indicated that the number of differentially expressed genes (DEGs) in the resistant interaction (3746) was much larger than that in the susceptible interaction (602). A great number of genes acting as intrinsic component of membrane, integral component of membrane, cell periphery and plasma membrance were remarkably enriched only in the resistant interaction, while the taurine and hypotaurine, phenylpropanoid pathway, plant-pathogen interaction and transcript factors were modulated in both interactions. This is the first study to examine genes expression patterns in a soybean genotype in response to invasion by a virulent and avirulent SCN population at the transcriptional level, which will provide insights into the complicate molecular mechanism of the nonhost resistant interaction.

## Introduction

Soybean (*Glycine max* (L.) Merr.) is an important crop that provides a valuable source of protein and oil all over the world [1]. However, soybean production is severely challenged by *Heterodera glycines* (soybean cyst nematode, SCN), which is one of the most devastating pathogens of soybean roots, causing losses of approximately 1.5 billion dollars annually in USA [2]. To date, the primary management for the control of SCN is breeding and growing resistant cultivars, which is the most economical and environment friendly solution [3]. The sources of resistance to SCN are identified as PI 88788, Peking and PI 437654, while another genotype Lee is susceptible cultivar.

SCN is a specialized plant-parasitic nematode, whose main hosts are leguminous plants including *Glycine max*, *Vigna angularis*, *Phaseolus vulgaris*, and *Pisumsativum*. While its poorly-parasitic hosts or non-hosts cover *Triticum aestivum*, *Hordeum vulgare*, *Zea mays*, *Oryza sativa*, *Nicotiana tabacum*, *Lycopersicon esculentum* and *Cucumis melo* [4]. However, the parasitism to the host is not immutable. For instance, one population of SCN can obtain the ability to reproduce on tomato by inoculation through several generations [5]. Coincidentally, our previous work demonstrated that SCN could also infect nonleguminous plants, among which, tobacco was first reported to be infected by SCN in Shandong, China. This special population is denominated as SCN_T_. It is worthwhile noticing that a nonhost resistant response occurred between soybean cultivar Lee and SCN_T_ [6], in which, although nematodes of SCN_T_ can reach the roots of soybean, plants are capable of preventing the growth, development and reproduction of nematodes in order to restrain the infection of nematodes to the roots [7]. The observation of this special population provides a valuable opportunity to explore the mechanism of the nonhost resistant response. While, Lee is proved to be the excellent host for HG 1.2.3.4.7 named SCN_S_. Based on our previous reports, there lies significant difference on parasitism of soybean between SCN_S_ and SCN_T_. However, SCN_S_ and SCN_T_ could not be differentiated from morphology, sequences of internal transcribed spacer (ITS) [6–9] and mitochondria *COⅠ* gene (data have not been showed). Nevertheless, the interaction mechanisms between the two populations and soybean roots remain elusive.

Identification and characterization of plant genes that differentially expressed are considered to be an effective and feasible solution to reveal molecular mechanism in this complex interaction [10]. High-throughput sequencing provides a means to scan differentially expressed genes involved in plant-SCN interaction. There have been increasing gene expression profiling researches of the SCN infection in plants for the last few years. The SCN-soybean compatible interactions have been performed using Affymetrix soybean whole-genome transcript array [11] or genome-wide association study [12, 13]. Besides, gene expression of soybean cultivar Peking infected by virulent and avirulent populations of SCN also have been assayed by Affymetrix soybean GeneChip [14] or single-end RNA-sequencing [15]. However, few studies through transcriptome sequencing have examined susceptible and resistant plant-nematode interactions in the nonhost plants.

To explore the molecular mechanism of SCN susceptible and resistant response of soybean cultivar Lee, it is essential to identify and characterize differentially expressed genes of soybean infected by the two populations of SCN. In this study the transcriptional profiles of both good-host and nonhost interactions of soybean-SCN were investigated using transcriptome sequencing. The result will provide potential candidate genes involved in the soybean-SCN nonhost resistant reactions.

## Materials and methods

### Plant germination and SCN inoculation

The soybean genotype Lee was used for the analysis, which exhibits susceptible to SCN_S_ but resistant to SCN_T_. Seeds were treated with 0.5% sodium hypochlorite solution for 15 min, followed by washes for three times with sterile water. Seeds were accelerated to germinate on moist sterile filter paper in dark at 27°C. After five days, seedlings were transplanted into plastic pots (7 cm × 5 cm × 8.5 cm) filled with sterile sands and irrigated the Hoagland nutrient solution. Three seedlings were placed in each pot, which were cultured under the condition of 16h light/8h dark cycle at 27°C with 50% relative humidity for four days.

The cysts of SCN_S_ and SCN_T_ were performed surface sterilization with 0.5% sodium hypochlorite solution for 1 min, followed by washing out using sterile water. The cysts were placed into 3 mM ZnSO_4_ solution in glass culture dish to incubate in dark at 27°C. The juvenile-stage 2 (J2) stage nematodes were collected and purified to be the suspension of 2000 J2/mL. Roots from one pot were inoculated with 1 mL suspension. While, roots of the control were inoculated with the same amount of sterile water. After 3-day inoculation, roots were collected into microfuge tubes, immediately frozen in liquid nitrogen and stored in refrigerator at -80°C. RNA samples were extracted from both control and inoculated roots using RNeasy plant mini kit (QIAGEN, Inc., Valencia, CA, USA) according to the manufacturer’s instruction. The single RNA pool of all treatments was subjected to RNAseq and qPCR analysis. Three biological replicate experiments were carried out, respectively.

### Transcriptome sequencing and analyses

The libraries were sequenced on an Illumina Hiseq platform 4000 and 125 bp/150 bp paired-end reads were generated. Three biological replicates were performed as above described for each inoculation. Differential expression analysis was performed using the EdgeR software. *P*-values were corrected for multiple hypothesis testing using the Benjamini-Hochberg false discovery rate (FDR). Genes with an adjusted *P*-value <0.05 were assigned as differentially expressed. The sequencing data were aligned to the reference genome using HISAT 2.0.4 software. In the hierarchical clustering, the FPKM (fragments per kb per million fragments) value was applied to represent the expression level of differentially expressed genes under different conditions. Gene Ontology (GO) enrichment analysis of differentially expressed genes was implemented by the GOseq R package. GO terms with corrected *P* value less than 0.05 were considered significantly enriched. KEGG analysis (http://www.genome.jp/kegg/) was performed using KOBAS software (http://kobas.cbi.pku.edu.cn/index.php) to test the statistical enrichment of differentially expressed genes in KEGG pathways.

### Quantitative Real-time PCR (qRT-PCR) Validation

qRT-PCR was performed to validate the results of transcriptome sequencing. A total of 12 genes were detected for qRT-PCR validation. RNA extraction was carried out as described above and cDNA synthesis (Takara, Japan) was conducted according to the manufacturer’s instructions. qRT-PCR was proceeded with SYBR Green PCR Master Mix (Takara, Japan) on a Real-time System (Jena, Germany). The actin gene (Genbank Accession: NM_001289231) was used as an internal control. All specific primers were list in Table 1. The relative expressions of 12 genes were calculated through the 2^-ΔΔ ct^ formula. Each sample was amplified by three biological replicates.

**Table 1:** Specific primers designed for qRT-PCR.

Gene id (Wm82.a1.v1)	Primer sequence (5'-3')	Product size/bp	Annealing temperature/°C
GLYMA15G13510	F: TTTTGACCCAACCACACCT R: TCACTCTGAAGCAAGCCCT	81	58
GLYMA02G46060	F: TCAAAGGGCAGACCACAG R: CAGGGCAACCTTGGAAAT	128	55
GLYMA18G13620	F: TGTTCAAGATGAATCAAGGGC R: AAATGGGAGATGGCTAAGGTC	331	58
GLYMA01G09280	F: GGTGTGGCAAAAGTTGTAGG R: GTTGGTGTTGGTGAAGATGG	330	58
GLYMA06G43940	F: AGACCCTACACCGTTTCACACAC R: ATCAACCAAAGACTCCAATCCAC	175	61
GLYMA06G45920	F: TTGGTGTTTCTCATTGTTCTTCA R: TGTTTCCTTATTTCTCCTTCTGT	379	58
GLYMA09G29330	F: TTGCCATTGTCATTTTCACCAC R: ACGCCTATAGCCATTCTCATCC	336	61
GLYMA05G25370	F: AACCTCCGCAAACCCTACCCT R: GATGGTGGTGGGTATTGTGCC	111	62
GLYMA14G39560	F: GAATCACCCATTTCCCACA R: ATGCCAAACAGTATCCTTCTCC	92	60
GLYMA17G12420	F: GACCGACTCATCATGCCCCT R: TTGCCCATCTCTTGTCCCAG	402	62
GLYMA04G01531	F: CCCAAAGCCAGAACTCCTC R: AGGAACCGATGAAGGGAAG	154	58
GLYMA16G01640	F: GCTAATGCGGAACACGAC R: AACTGCGACAGCAACCCT	338	58

## Results

### Quality assessment of sequencing data

The three biological replicates of each treatment were sequenced respectively. Q30 standards of each sequencing data were all more than 90% and GC percent of each sequencing data were 35–65%, which indicated sequencing data presented better quality and could be used for bioinformatic analysis. The number of raw reads ranged from 42–54 million. Approximately 91–92% of the filtered reads which aligned uniquely to the reference genome were performed to further analysis (**Table 2**). All of the clean data were deposited in the Short Read Archive database of NCBI web site under the Accession Number PRJNA494484.

**Table 2:** Sequences Statistics of raw reads for G*lycine max* transcriptome infected by SCN_S_ and SCN_T_ population 3 days post inoculation and non- inoculation (CK).

Line	Replicates	Raw reads	Clean reads	Uniquely mapped	Aligment (%)
CK	1	48051760	47175578	43483578	92.17
	2	49537316	48584212	44686238	91.98
	3	45497900	43733308	40115127	91.73
SCN_S_	1	42630430	41102714	37835492	92.05
	2	47096186	46233940	42550270	92.03
	3	54873922	53910248	49655205	92.11
SCN_T_	1	48744484	47734056	43467452	91.06
	2	45308140	43975692	40578153	92.27
	3	53305074	51755406	47427365	91.64

### Screening of differentially expressed genes (DEGs)

**Fig 1:**
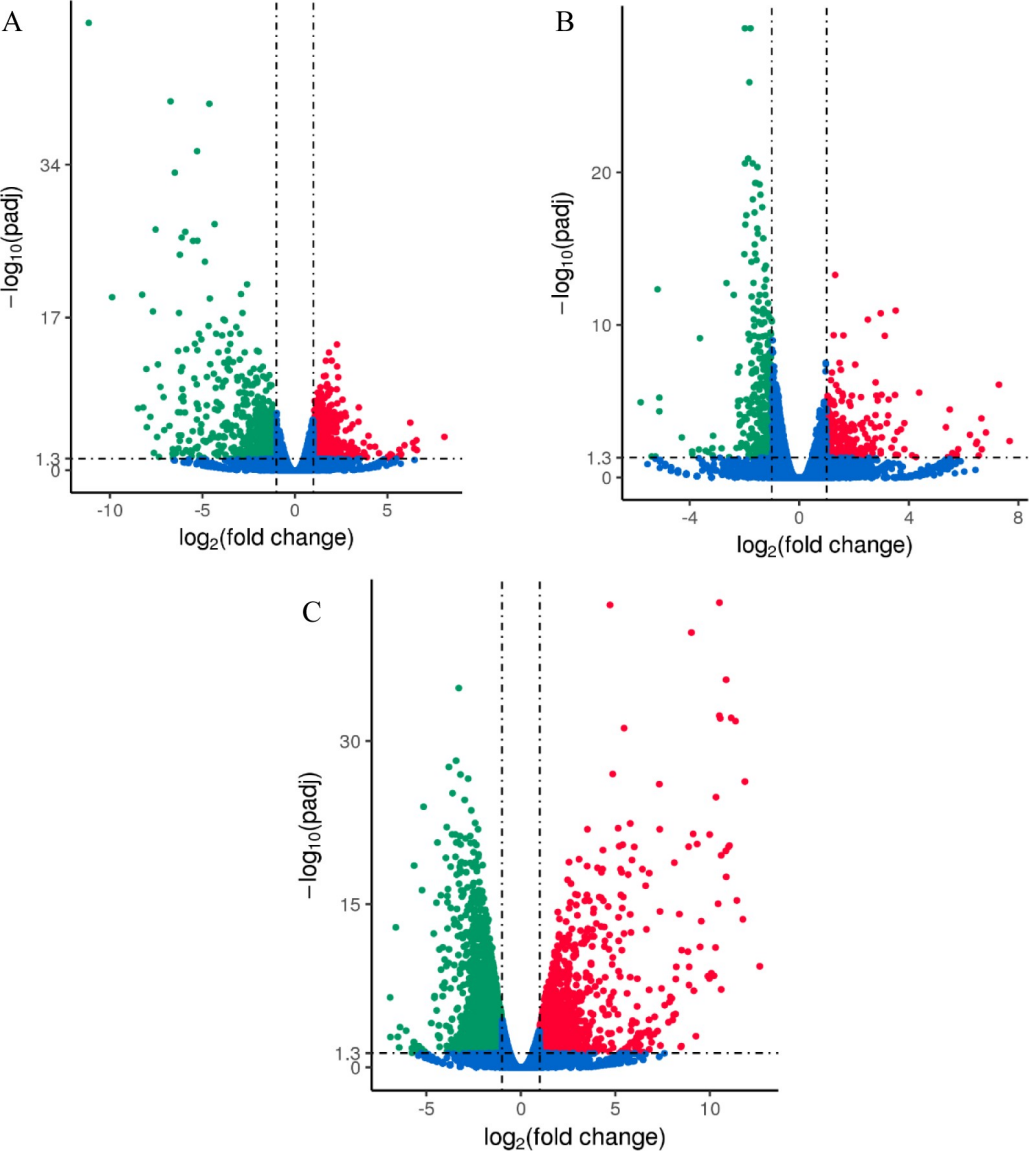
Sequences Transcriptome analysis of gene regulation in susceptible and resistant interactions. (A) soybean roots infected by SCN_S_ vs. soybean roots infected by SCN_T_ (SCN_S_ vs. SCN_T_). (B) soybean roots infected by SCN_S_ vs. soybean roots non-infected (SCN_S_ vs. CK). (C) soybean roots infected by SCN_T_ vs. soybean roots non-infected (SCN_T_ vs. CK). The x-axes indicate fold change values (*P*<0.05) and the y-axes represent the statistical significance of differences of gene expression. DEGs are shown in red and green dots indicating up-regulated and down-regulated genes respectively.

**Fig 2:**
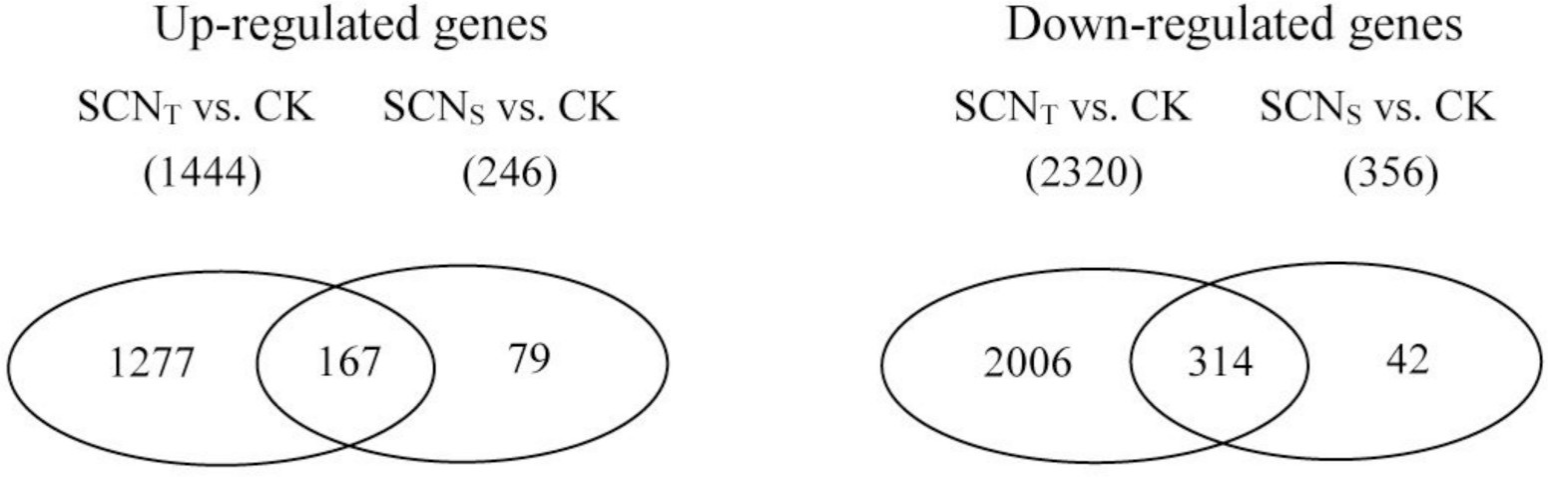
Venn diagrams presenting common and unique DEGs in resistant (SCN_T_ vs. CK) and susceptible (SCN_S_ vs. CK) interactions. In total, 481 genes were common between the two interactions.

Transcriptome analysis of both SCN_S_ and SCN_T_ infecting soybean roots compared with non-infected samples were visualized by the volcano plot. The criteria of differential gene screening was adjusted *P*-value <0.05. The DEGs were screened according to the log2 fold change and significant level. As shown in the volcano plot, there were 1713 DEGs between SCN_S_ and SCN_T_, of which 883 genes were up-regulated and 830 were down-regulated (Fig 1A). In the compatible interaction, compared with non-infected samples, 602 DEGs were detected, among which transcriptional levels of 246 genes were up-regulated, with 356 being down-regulated (Fig 1B). However, there were more DEGs tested in the resistant interaction, which presented 1444 up-regulated DEGs and 2320 down-regulated ones (Fig 1C). Despite different pathogenicity of SCN_S_ and SCN_T_ to soybean, soybean infected by SCN_S_ and soybean infected by SCN_T_ shared 479 DEGs (166 DEGs in up-regulation and 313 DEGs in down-regulation) compared with soybean non-infected (Fig 2). Furthermore, no genes were detected up-regulated in the resistant interaction and down-regulated in the susceptible interaction, while expression of one gene (Glyma06g15030) was downregulated in the resistant interaction but upregulated in the susceptible interaction.

### Hierarchical clustering of DEGs

**Fig 3:**
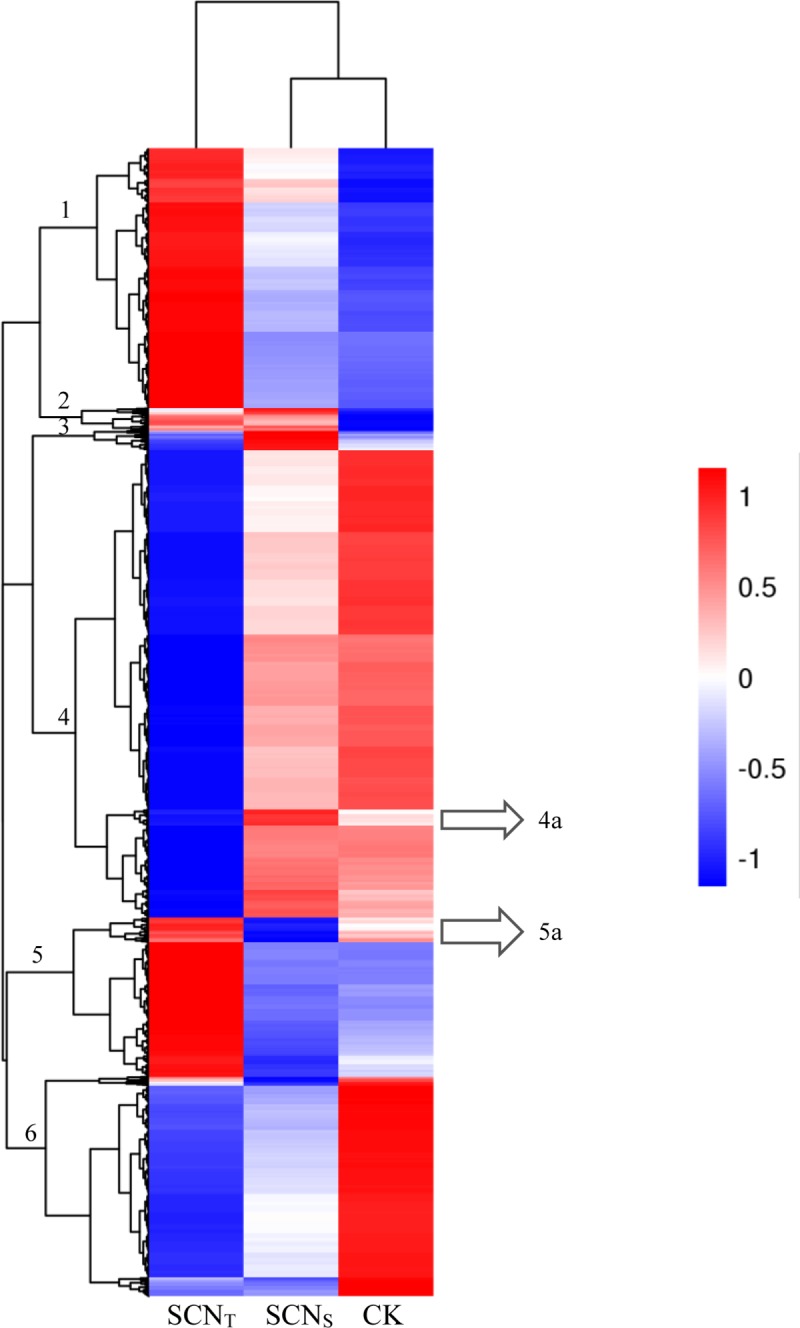
Hierarchical clustering of DEGs. Each column represents a sample and each row means a gene. 4182 genes were regulated by SCN infection. SCN_T_: gene expression of soybean roots infected by SCN_T_; SCN_S_: gene expression of soybean roots infected by SCN_S_; CK: gene expression of soybean roots non-infected. Red color: up-regulation; Blue color: down-regulation.

The hierarchical clustering obviously exhibited that soybean roots had different responses to different SCN populations infection. According to the sample clustering, the patterns of SCN_S_ and CK were consistent, which were clustered together. Whereas, the sample of SCN_T_ presented opposite pattern to the other two samples. Based on the gene clustering, DEGs were clustered into six major clades (Fig 3)

Clade 1: almost all of DEGs had a high expression level in SCN_T_, with low expression in SCN_S_ and CK.

Clade 2: genes whose expression was decreased in CK were slightly up-regulated in SCN_S_ and SCN_T_.

Clade 3: gene expressions mainly downregulated in SCN_T_ were upregulated by SCN_S_, with almost no change in CK.

Clade 4: the trend of genes expression was opposite to Clade 1 with exception of a subbranch 4a, in which the transcriptional levels of genes were notably enhanced in SCN_S_, while these genes had no change in CK.

Clade 5: the pattern of genes transcript was as similar as Clade 1. In addition, there was an interesting subbranch 5a, in which DEGs were significantly decreased by SCN_S_ but were slightly up-regulated by SCN_T_ and CK.

Clade 6: genes possessed high expression level in CK but low level in SCN_S_ and SCN_T_.

### Gene ontology (GO) analysis of DEGs

**Fig 4:**
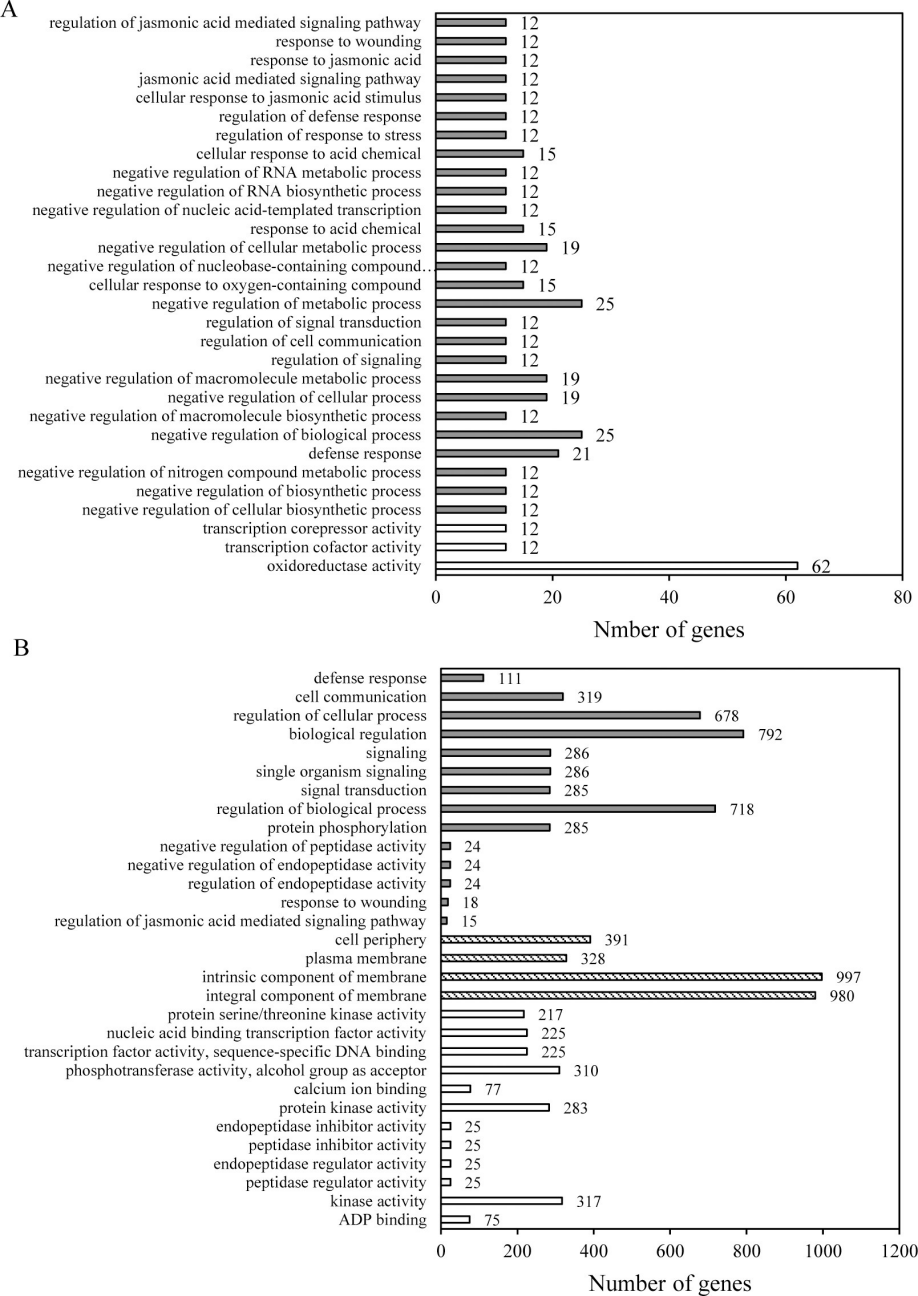
**Classification of DEGs based on gene ontology in the susceptible interaction (A) and resistant interaction (B).** The number of DEGs categorized under biological process in soybean roots infected by SCN_S_ and SCN_T_, respectively, were presented as grey bars. Genes belonging to molecular function were represented as light bars. In addition, the dashed bars mean the DEGs of cellular component.

The 30 GOterms which were the most significant enrichment in the susceptible and resistant interactions were exhibited in Fig 4 according to gene ontology. The 30 most abundantly represented terms in the susceptible reaction were shown in Fig 4A. the significant enrichment focused on molecular function and biological process, in which the enrichment degree of the GOterm representing regulation of jasmonic acid mediated signaling pathway (GO:2000022) was the most remarkable. A notable number of genes (33 up-regulated and 29 down-regulated) functioning as oxidoreductase activity were assembled under the GO term (GO:0016491).

However, the enrichment result of the resistant interaction (Fig 4B) was distinctly different from that of the susceptible interaction. It was noteworthy that the cellular component was significantly enriched, in which there was the most prominent number classified as intrinsic component of membrane (GO:0031224) and integral component of membrane (GO:0016021). There were three common GOterms between susceptible and resistant interaction, including jasmonic acid mediated signaling pathway (GO:2000022), response to wounding (GO:0009611) and defense response (GO:0006952), among which, the GO term denoting defense response was significantly enriched.

### KEGG analysis of DEGs

**Fig 5:**
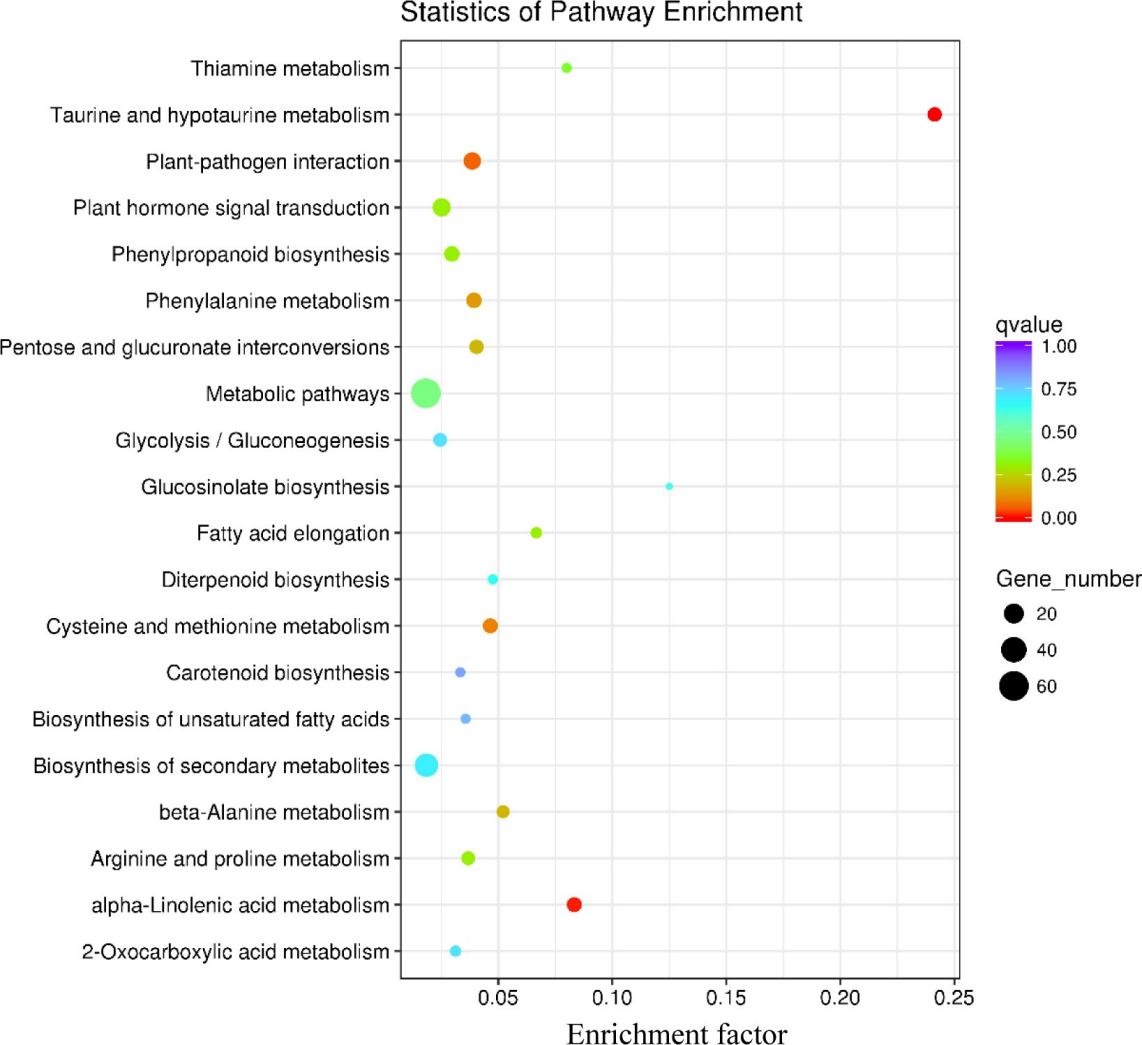
KEGG enrichment analysis of genes significantly regulated in the susceptible interaction. Enrichment factor means the ratio of the number of DEGs enriched in this pathway to that of annotated genes. The value is significant at *q* < 0.05.

**Fig 6:**
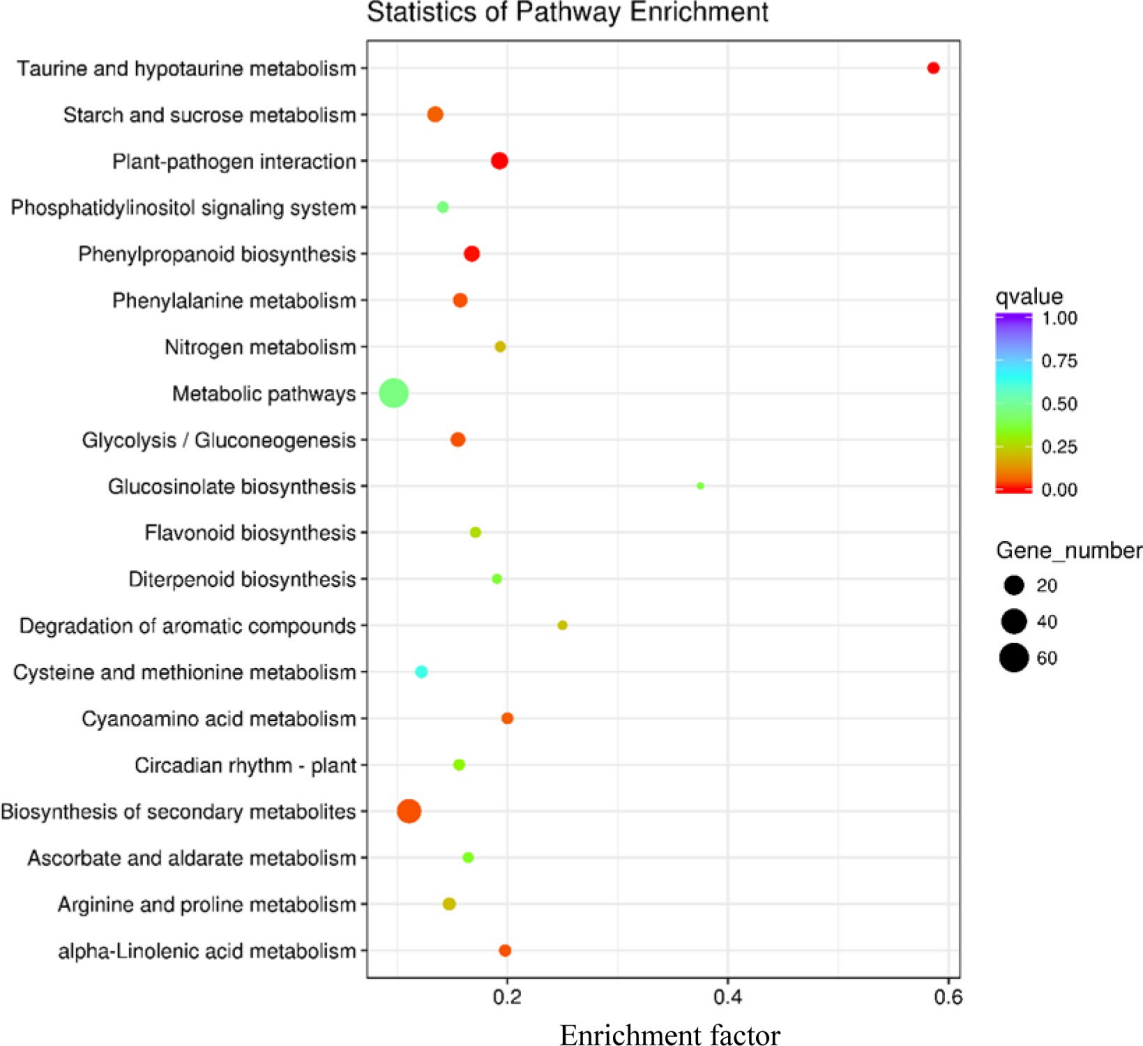
KEGG enrichment analysis of genes significantly regulated in the resistant interaction. Enrichment factor means the ratio of the number of DEGs enriched in this pathway to that of annotated genes. The value is significant at *q* < 0.05.

The KEGG pathway enrichments of DEGs in the susceptible and resistant interactions were shown in Figs 5 and 6, respectively. The result demonstrated that both susceptible and resistant interactions shared the common pathways, which included taurine and hypotaurine metabolism, plant-pathogen interaction, phenylalanine metabolism, phenylpropanoid biosynthesis, metabolic pathways, glycolysis, glucosinolate biosynthesis, diterpenoid biosynthesis, cysteine and methionine metabolism, biosynthesis of secondary metabolites, arginine and proline metabolism and alpha-linolenic acid metabolism. Besides, there were a large number of genes involved in metabolic pathways of both interactions. The most significant enrichment in the susceptible interaction was taurine and hypotaurine metabolism, followed by alpha-Linolenic acid metabolism and plant-pathogen interaction (Fig 5). Similarly, in the resistant interaction, taurine and hypotaurine metabolism was also the most notably enriched (Fig 6). Interestingly, pathways involved in vitamin biosynthesis and metabolism also appeared in the two interactions. In the metabolic pathways, Carbohydrate metabolism, Lipid metabolism and Nucleotide metabolism were the three most abundantly represented pathways with 61 DEGs in the susceptible reaction and 325 DEGs in the resistant reaction. Thiamine metabolism and carotenoid biosynthesis occurred only in the susceptible interaction (Fig 5), with ascorbate metabolism uniquely in the resistant interaction. In addition, flavonoid biosynthesis and nitrogen metabolism were exclusively enriched in the resistant interaction (Fig 6).

### qRT-PCR analysis

As shown in Table 3, the qRT-PCR data were correlated with transcriptome sequencing result. Although the expressions of genes were different in transcriptome sequencing and qRT-PCR, the trends of genes change were consistent.

**Table 3:** The validation of transcriptome sequencing data using qRT-PCR.

Sample	Gene id (Wm82.a1.v1)	Name	Fold change (log 2)	
			RNA-seq	qRT-PCR
SCN_S_	Glyma15g13510	peroxidase (GMIPER1)	6.23	7.21
	Glyma02g46060	Zinc finger	5.80	3.59
	Glyma18g13620	glutathione S-transferase (GSTF12)	3.47	5.28
	Glyma01g09280	MYB39	-4.29	-3.48
	Glyma06g43940	O-methyltransferase	-2.19	-4.12
	Glyma06g45920	peroxidase (PC7)	-2.29	-3.75
SCN_T_	Glyma09g29330	proteinase inhibitor I3	10.59	8.57
	Glyma05g25370	polygalacturonase inhibitor	5.20	6.31
	Glyma14g39560	hsp20	3.19	2.76
	Glyma17g12420	PTR FAMILY 6.2	-6.61	-4.22
	Glyma04g01531	TIFY 10A	-3.94	-2.19
	Glyma16g01640	pectinesterase 3	-1.02	-1.63

## Discussion

There has been increasing research mainly focusing on different soybean genotypes which displayed resistant or susceptible to the same SCN population [10–12, 16]. However, these studies were not fully able to take into account part of DEGs screened from the above studies possibly caused by different genotypes. In spite of a microarray analysis of the same soybean genotype infected by *Heterodera glycines* with compatible and incompatible responses, the resistant cultivar Peking was employed in this work to investigate plant defense responses [14]. Although microarray analysis was conducted in SCN-susceptible soybean [17–19], the differences between susceptible and resistant responses in the SCN-susceptible variety remain unknown. Based on the above studies, we conducted the comparative transcriptome sequencing to examine SCN-susceptible soybean genes involved in susceptible and resistant responses to invasion of different SCN populations, which would provide an insight into soybean distinct responses to SCN populations of significantly different pathogenicity. As a result, genes related to the interaction in the soybean cultivar Lee could be screened to reveal infection mechanism of SCN. This is the first study to examine genes expression patterns in a SCN-susceptible soybean genotype in response to invasion by highly and weakly pathogenic SCN populations to describe a nonhost resistance response in soybean against nematodes at the transcriptional level using Illumina 2000 sequence technology.

### More DEGs identified in incompatible interaction than those in compatible interaction

Through comparative transcriptome analysis, more DEGs were identified in the resistant interaction (3764) than those in the susceptible interaction (602) after 3 dpi, which was similar to the report that 6043 genes were significantly regulated in incompatible interaction with 3309 genes in compatible interaction at 3 dpi [14]. However, the number of DEGs was striking different between the above results, the reason for which might be that different soybean varieties were applied. The variety (Lee) susceptible to SCN was used in our research, while the resistant genotype (Peking) to SCN was applied in that study. The SCN-resistant genotype might mobilize much more differentially expressed genes to resist SCN infection. Another reason we speculated could likely be the time of infection, which was supported by the above study that more DEGs appeared in the compatible interaction than those in the incompatible interaction at 8 dpi [14].

Despite significantly different pathogenicity of SCN_S_ and SCN_T_ to soybean, both reactions shared 479 DEGs (166 DEGs in up-regulation and 313 DEGs in down-regulation) (Fig 2), which demonstrated there were some common pathways of soybean response to different SCN populations. For instance, up-regulated DEGs were commonly enriched under Taurine and hypotaurine metabolism and Cysteine and methionine metabolism, while alpha-Linolenic acid metabolism and Plant-pathogen interaction were the most two significant enrichments in down-regulation.

### The cellular component only remarkably enriched in the incompatible reaction

The interaction between plants and pathogens is highly complex. A primary challenge for pathogens is to break the physical barrier of host cell walls [20]. Similarly, nematodes need to break down cell walls of their host to obtain nutrition for their growth. On the other hand, plants have evolved to recognize pathogens and remodel the cell wall integrity to prevent the disease [21]. During this process, various changes can be triggered in host cell walls to defense nematode attack.

For instance, the obvious difference of our gene ontology analysis between the two interactions was that the cellular component was not remarkably enriched in the susceptible interaction, while a great number of differentially expressed genes categorized under cellular component were detected in the resistant interaction (Figs 5 and 6). In cellular component, DEGs were mainly assigned to intrinsic component of membrane, integral component of membrane, cell periphery and plasma membrance with 997 (26.49%), 980 (26.04%), 391 (10.39%) and 328 (8.71%) unigenes, respectively. Similar observations were also identified in the resistant interaction between a resistant genotype and HG type 2.5.7. [13].

Furthermore, this result also indicated that when soybean invaded by different pathogenic SCN populations, the cellular component of soybean was upregulated to exhibit different transcriptional levels. To be specific, SCN_S_ possess a high pathogenicity to the soybean cultivar Lee, in which the transcript of cellular component showed a slight change. However, SCN_T_ infecting soybean caused more abundance of the cellular component at the transcriptional level, which implied that the cellular component might play an essential part to defense SCN_T_ attack, resulting into rendering Lee resistant to SCN_T_. Similar responses were also observed in resistant soybean cultivar after infection by *Heterodera glycine* [10, 16] or *Meloidogyne incota* [22]. Consequently, the resistant reaction could occur possibly due to amounts of genes involved in cell wall remodeling at the early infection, but this hypothesis needs further verification.

### The taurine and hypotaurine was the most notably enriched in both interactions

The taurine and hypotaurine are amino acid derivatives in many eukaryotes [23], which induces an efficient detoxifying enzymatic action and scavenging singlet oxygen [24]. However, in plants, the potential roles of them have not been extensively studied in higher plants to date. The analysis of taurine *in vitro* was identified as anti-stress agent in tomato [25], while little was known about it function *in vivo*. Recently, Zhao et al. showed that the taurine and hypotaurine metabolism was the top pathway in resistant and susceptible tomato infected by *Fusarium oxysporum* [26]. Similar results were also found in our study that this metabolism was the most notably enriched in both interactions (S1 Table), implying that this pathway was the common one implicated in the soybean-SCN interaction in soybean cultivar Lee.

Overall, 24 genes were differentially expressed in both interactions. Genes encoding plant cysteamine dioxygenase and glutamate decarboxylase were notably enhanced. In contrast, genes encoding a glutamyl transpeptidase had a dramatical reduction. The first two enzymes mentioned synthesize taurine and hypotaurine, respectively, while the latter exhibits a decomposition reaction of taurine. The above results implied that when soybean attacked by SCN, the contents of taurine and hypotaurine were regulated by the three enzymes at the transcriptional level. Nevertheless, this pathway was not enriched in other researches on susceptible soybean during infection by SCN [18, 27]. The inconsistent result might sound reasonable that different susceptible soybean genotypes resulted in differences response to SCN. Therefore, further examinations should be carried out to explore the functions of taurine and hypotaurine in different soybean genotypes during SCN invasion.

### Genes involved in the phenylpropanoid pathway exhibited uniquely in susceptible soybean after SCN infection

The plant employs a number of secondary metabolites in the defense against the pathogen [28]. It has already been verified that phenylpropanoids are part of the chemical defense system of plants against parasitic nematodes [29].

In our study, the phenylpropanoid pathway was enriched in both interactions (S2 Table). The previous reports also identified differentially expressed genes in the phenylpropanoid pathway [11, 30, 31], indicating this pathway possibly plays a critical role in soybean roots during SCN invasion. However, our transcriptome sequencing results showed that soybean roots infected by the SCN_T_ population presented 51 genes (9 up-regulated and 42 down-regulated) differentially expressed involved in this pathway. Among them, genes encoding key enzymes of phenylpropanoid biosynthesis, such as O-methyltransferase, 4-coumaryl CoA ligase and phenylalanine ammonia-lyase (PAL) were dramatically decreased, which was inconsistent with resistant soybean infected with SCN at 3 dpi [10]. Besides, transcript levels of the three enzymes showed a slight decrease in the susceptible reaction. On the contrary, three genes described above were notably increased in other reports [18, 11]. This contradiction might be explained that different soybean cultivars applied in researches, which was well documented in the previous work. The transcriptional abundances of genes encoding PAL and 4-coumaryl CoA ligase, along with activities of the two enzymes were significantly increased in resistant but not in susceptible soybean genotypes after SCN infection [32]. The result implicated that resistant and susceptible cultivars might regulate different members of the multigene family involved in the phenylpropanoid pathway in soybean. Therefore, further investigations need to be carried out to reveal the precise mechanisms of this pathway in resistant and susceptible soybean cultivars.

### Plant-pathogen interaction was notably enriched

Similar to published reports [16, 33], our data identified that plant-pathogen interaction (PPI) was enriched, with 13 genes involved in the susceptible interaction and 65 genes in the resistant interaction (S3 Table). One type of genes involved in PPI was plant resistance genes, which could recognize pathogen and activate immune response to inhibit pathogen proliferation [34, 35]. In the last few years, lots of plant resistance genes have been identified and characterized from model plants [36–38]. These proteins contain a nucleotide binding (NB) and leucine-rich repeat (LRR) domain, referred to as the NB-ARC domain [39]. The similar finding was acquired in our study. For instance, four genes containing the NB-ARC domain (Glyma01g35120, Glyma09g34360, Glyma01g01420 and Glyma06g46830) were notably enhanced in soybean roots infected by SCN_T_ but showed no significant change in those infected by SCN_S_ compared with the control, which implied that these genes might play an essential part in soybean to stimulate resistant response to SCN_T_ attack. Further researches need to be performed to explore its possible role in mediating soybean resistance to SCN_T_.

In addition, as is well-known that pathogenesis-related protein 1 (PR1) is the most abundantly produced protein against pathogen attack in plants [40]. The similar role of PR1 were also characterized in soybean response to SCN in the previous report, in which, the *PR1* gene was the most highly upregulated in both compatible and incompatible reactions using Perking (resistant to SCN) as plant material [14]. What was inconsistent was that in our study, the transcript of *PR1* was not significantly regulated in the susceptible reaction. The same result as ours was obtained in tomato tissues infected by *Meloidogyne incognita* [41]. In the susceptible tomato, changes in the *PR1* gene upregulated by the nematode invasion were insignificant. The reason for the different results might also be soybean cultivars exhibiting susceptible or resistant responses to SCN. Therefore, the slight change of *PR1* might be one of the causes of the nematode disease in susceptible plants undergoing the compatible reaction.

### Transcription factors (TFs)

There are increasing evidences demonstrating that TFs have been identified to participate in soybean defense response to pathogens [42–43]. Zinc finger proteins are a superfamily involved in many aspects of plant growth and development, as well as an important regulator in plant responses to abiotic and biotic stresses [44]. In our study, we identified 124 genes encoding Zinc finger proteins differentially expressed in the resistant interaction, 73 (58.87%) of which were strongly upregulated after SCN_T_ infection (S4 Table). We classified 73 proteins into nine types (RING-type, C2H2-type, PHD-type, MYND-type, CCCH-type, Dof-type, CHY-type, SWIM-type and C5HC2-type). Expressions of DEGs encoding Zinc finger C2H2-type proteins were also significantly increased in *Arabidopsis* roots infected by *Meloidogyne javanica* [45]. Besides, our results showed that RING-type and C2H2-type contained 35 and 10 DEGs, respectively, representing the top two largest SCN_T_-upregulated Zinc finger types. Notably, two RING-type DEGs (Glyma02g46060 and Glyma09g26100) with the most remarkable enhancements (24.88- and 19.02-fold) in the resistant reaction showed only 5-fold up-regulation and no significant change, respectively, in the susceptible reaction. These results suggested that Zinc finger family genes might positively regulate downstream target genes to establish resistance to SCN_T_ at the transcriptional level. In addition, *ERF* performing the role in plant defense responses to various stresses [46] were also notably upregulated in the resistant interaction. It provided the evidence that *ERF* genes as one of the major branches participating JA signaling pathway were also upregulated in soybean under *Heterodera glycines* infection [13].

## Conclusion

This is the first report describing genes profiles of SCN-susceptible soybean involved in resistant and susceptible responses. Our study revealed specific regulations of genes involved in the nonhost resistant reactions to SCN infection at the transcriptional level in which, a number of transcripts with different accumulations exhibited distinct responses to different SCN populations. Functional confirmations of significantly regulated genes can be performed by overexpressing or silencing them in soybean. Overall, comparative transcriptome analysis provides insights into the complicate molecular mechanism of the SCN-susceptible soybean served as both good-host and nonhost interactions.

## Supporting information

S1 Table List of DEGs involved in taurine and hypotaurine in both susceptible and resistant response. (XLS)

S2 Table List of DEGs involved in phenylpropanoid pathway in both susceptible and resistant response. (XLSX)

S3 Table List of DEGs involved in plant pathogen interaction in both susceptible and resistant response. (XLSX)

S4 Table List of transcriptional factors with significant transcriptional abundance in both susceptible and resistant response. (XLSX)
